# Ligation of the Femoral Artery, Twenty Days after Being Wounded

**Published:** 1863-02

**Authors:** R. B. Treat, Amos S. Jones

**Affiliations:** Janesville, Wisconsin; Janesville, Wisconsin


ARTICLE VI.

### LIGATION OF THE FEMORAL ARTERY, TWENTY DAYS AFTER BEING WOUNDED.

BY R. B. TREAT, M.D., and AMOS S. JONES, M.D., Janesville, Wis.

Reported for the Chicago Medical Examiner.

On the 2d of Jan., 1861, we were requested by Dr. COVERT, of Clinton, Wis., to see with him Alfred Lee of Manchester, Ill. From Dr. COVERT, the attending physician, we obtained the previous history of the case as follows:—

On the 13th of Dec., 1860, while said Lee was standing by a bench cutting pork with a large dirk-pointed knife, with the edge and point inclined obliquely downward and toward him, the point of the knife glanced from the meat, and forcibly struck his leg, over the junction of the femoral and popliteal arteries, inflicting a wound an inch in length and deep enough to wound the artery. Profuse hemorrhage immediately ensued, causing almost fatal syncope. The coagula, and a compress, and roller, applied by a neighbor, arrested the hemorrhage almost entirely; and after an hour and a-half his consciousness returned, with scarcely perceptible pulse, skin perfectly blanched, and almost total prostration. Dr. COVERT first saw the case nine hours after the accident, and did not deem it advisable to disturb the wound. He directed tonics with a little stimulant, and occasionally an aperient, with easily digested and nourishing diet, and rest and quiet in a recumbent position. On the 19th, he was called in haste to see Lee, by a messenger, who stated that he was bleeding to death. (Lee lived five miles from Dr. COVERT.) A travelling quack, (and we have several in this vicinity, besides resident quacks,) had seen the case, and promised to fix the wound all right with puff-ball, &c. The hemorrhage was again very profuse, occasioned by removing the compress and roller, fussing with the puff-ball, &c., on the part of the quack. He, however, succeeded by puff-ball and pressure in checking the bleeding partially. This time long continued syncope was produced; and it was surprising that he did not die. When Dr COVERT arrived, he applied a firm compress and roller, which controlled the hemorrhage most of the time, and occasionally a little turpentine was poured on the compress. Thus matters passed along several days longer. Dr. COVERT saw the case again on the 22d, and desired counsel; and an old physician from Beloit was obtained. He was informed of all the history of the case to that time; and from it, and the appearance he saw, he decided that no artery was wounded, and advised the continuation of Dr. COVERT'S previous treatment. The case passed along several days longer with occasional hemorrhage, the leg swollen in the region of the wound, the patient unable to move in bed, exhibiting an unusual tenacity to life; iron, quinine, and other tonics, and sometimes stimulants with appropriate diet were given him; but the wounded artery would not heal!

Dr. COVERT, not satisfied that the patient and wound were doing well, desired other counsellors, and sent for us to visit the case, which we did by the first train. We found the man extremely reduced. He could not communicate above a faint whisper, could not raise his hand in bed, and presented the most cadaverous appearance we ever beheld in a living human being. The leg (right) was edematous, but not very badly swollen, except in the vicinity of the wound. The temperature was much lower than natural, yet did not require the constant application of artificial heat. The pulse was scarcely perceptible at the wrist, and was wirey and quick. The body was very much emaciated. The whole region about the wound, for the space of six inches in every direction from its centre, was perfectly infiltrated and firmly impacted with pus, serum, and coagula, presenting a very disagreeable appearance, and not at all promising in regard to saving life, and much less the limb. He and his friends implored us to save his life and limb, if possible. We told them the chances were strongly against him, and that most probably he would die in the operation, either of ligating the artery or amputating the leg. There seemed to us no doubt but that the femoral artery was either wounded or severed. From the fact of the circulation being so good, under the circumstances, in the leg, below the wound, we judged that if he survived the operation of tying the artery, his leg might possibly be saved.

We decided to ligate the artery just above the wound, so that in case amputation should be required in future, we might have a sound artery to tie, and also, which was of more immediate importance, that the circulation in the leg, below the wound, would be as little as possible interfered with. It was now dark; and we began and completed the operation with the illumination produced by two of the poorest kind of dipped tallow candles:—The patient was moved to the edge of the bed,—leg a little flexed, and held by an assistant; no anesthetics were administered. The artery was compressed over the pubis by assistants, when we proceeded to clear out the wound as much as possible; and removed over a pint of coagula and other matter from the wound and its environs before making any incision. We then made an incision four inches in length, extending from the wound upward or toward the body, and in depth to the outer edge of the sartorius muscle. Then followed an interesting (?) search for the femoral artery, such an one as we desire not to be obliged to make again. The tissues were so changed by abnormal deposit, destruction, and discoloration, the incision so deep, the oozing so constant, the circulation so feeble, and the light so poor, that in the discovery and ligation of the femoral artery at this point, about two inches above the wound; and, under the existing circumstances, we congratulated ourselves that we had performed one of the most difficult operations of surgery. We had no medical assistant besides Dr. COVERT. The compression of the artery was no light task, and was a poor remedy for numb fingers and thumbs.

But at last success crowned our efforts. The patient complained very little and scarcely moved a muscle during the whole operation, and bore it with fortitude and patience almost superhuman. Very little blood was lost during the operation; and after dressing the wound, and giving him a drink of whisky, he felt extremely well. After giving directions in regard to medicine and diet, we departed for home. We saw no more of our patient until about six weeks afterwards, he came into our office in tolerable health and strength, full of gratitude for our benefactions. We saw him again in two or three months, in perfect health, and looking tough and rugged. There was no lameness or weakness of the leg; and he is alive and sound now, so far as we know. His age was about 27, is a farmer, and was then unmarried.

We give below the principal treatment and condition after the operation, as given by Dr. COVERT:—

January 4th.—Some diarrhœa; pulse very feeble; wound very offensive; sloughing extensive. Gave geranin for diarrhœa. For tonics.—Ferri. mur. tinct., quina., and nourishing diet, with occasionally a stimulant of brandy. The wound was washed with a solution of potas. chlo. and hydrastin, and dressed several times a day, also wet with arnica liniment. This treatment mainly was continued to the time of his recovery.

The ligature came away on the 15th day after the operation.

The wound was healed by the 20th day. Feb. 4th.—Began to use his leg in locomotion. In a week or two after, was walking about with perfect facility. There was very little difference in the appearance of his legs three months afterwards, except the cicatrix.

We have dwelt thus lengthy, and, perhaps, tediously, on the report of this case, because we regarded it a remarkable recovery after such profuse hemorrhage, and the lapse of so long a time from the accident to the ligation of the artery; but all praise is due to the vis medicatrix nature, analeptics, patience, and hope.

JANESVILLE, WIS., *Feb. 26th*, 1863.

